# Glial activation among individuals with neurological post-acute sequelae of coronavirus disease 2019: A positron emission tomography study of brain fog using [^18^F]-FEPPA

**DOI:** 10.1016/j.bbih.2025.100945

**Published:** 2025-01-16

**Authors:** Sean A.P. Clouston, Paul Vaska, Tesleem Babalola, John Gardus, Chuan Huang, Nicola Soriolo, Ashley Fontana, Christine DeLorenzo, Ramin Parsey, Benjamin J. Luft

**Affiliations:** a Program in Public Health, Stony Brook University, Stony Brook, NY, 11794, USA; b Department of Family, Population, and Preventive Medicine, Stony Brook University, Stony Brook, NY, 11794, USA; c Department of Radiology, Stony Brook University, Stony Brook, NY, 11794, USA; d Department of Biomedical Engineering, Stony Brook University, Stony Brook, NY, 11794, USA; e Department of Psychiatry, Stony Brook University, Stony Brook, NY, 11794, USA; f Department of Radiology and Imaging Sciences, Emory University, Atlanta, GA, 30322, USA; g Department of Biomedical Engineering, Emory University and Georgia Institute of Technology, Atlanta, GA, 30322, USA; h Department of Medicine, Stony Brook University, Stony Brook, NY, 11794, USA

**Keywords:** Post-acute sequelae of COVID-19, Respiratory infection, Glial activation, Translocator protein, Essential Workers, Positron emission tomography, FEPPA, Brain Fog, COVID-19

## Abstract

**Background:**

This study examined the regional distribution of glial activation in essential workers with neurological post-acute sequelae of coronavirus disease 2019 (COVID-19) infections (N-PASC).

**Methods:**

We injected ≤185 MBq of [^18^F]-FEPPA as an intravenous bolus and positron-emission tomography over 2 h. To measure distribution volume (V_T_) we recruited 24 essential workers (14 N-PASC, 10 Never-COVID-19 Controls, of whom 22 successfully placed arterial lines). Individuals with low binding affinity were excluded from this study, and V_T_ was adjusted for translocator protein genotype. Analyses that passed the false discovery rate are reported.

**Results:**

Participants at midlife survived mild to moderate COVID-19 without hospitalization but reported onset of post-acute sequelae of COVID-19 (PASC) for, on average, 22 months before undergoing neuroimaging. Hippocampal V_T_ was higher (V_T_ = 1.70, 95% C.I. = [1.30–2.21], p = 0.001) in participants with persistent brain fog after COVID-19, reflecting an increase of 10.58 mL/cm^3^ in V_T_ (area under the receiver-operating curve, AUC = 0.95 [0.85–1.00]). At a cutoff of 10.6, sensitivity/specificity/accuracy were 0.88/0.93/0.91.

**Conclusion:**

The results from this study imply that neuroimmune response is a distinct and identifiable characteristic of brain fog after COVID-19. Results suggest that [^18^F]-FEPPA could be used to support N-PASC diagnosis.

## Highlights

• Neurological symptoms are a central feature of post-acute sequelae of COVID-19.
• To date, there are no protocols for monitoring brain health in PASC.
• Glial activation on positron-emission tomography achieved excellent accuracy.
• At a cutoff of 10.6, sensitivity/specificity/accuracy were 0.88/0.93/0.91.

## Graphical abstract

**Figure:**
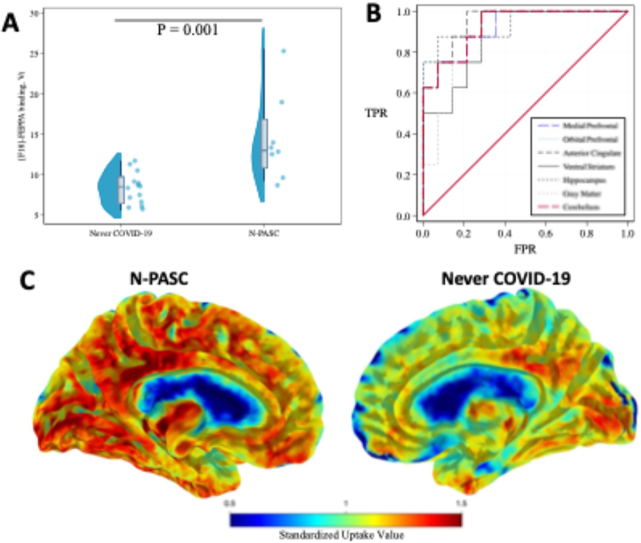

## 1 Background

Severe and acute respiratory syndrome coronavirus-2 (SARS-CoV-2) is an airborne ribonucleic acid virus that is highly contagious in humans. Among those who reported developing coronavirus disease 2019 (COVID-19), studies report changes in the cerebral connectome consistent with neuroinflammation among hospitalized patients with severe COVID-19 (Rau et al., 2022) that appears not to return to normal in some patients after recovery (Huang et al., 2022). Coincidentally, while most infected individuals only develop an acute syndrome, 20% of unvaccinated individuals (Yoo et al., 2022) and 10% of vaccinated individuals with breakthrough cases of COVID-19 (Thaweethai et al., 2023) develop Post-Acute Sequelae of Coronavirus Disease (PASC) that can persist for years after infection and is strongly associated with the severity of acute disease even in midlife (Lhuillier et al., 2022). Despite the widespread availability of vaccines, the risk of PASC has not been eradicated (Kahlert et al., 2023) and PASC is a growing cause of disability globally (Cai et al., 2024). Thus, there remains a need to better understand the significance of neuroimmune reactions in PASC (Mantovani et al., 2022).

Neurological symptoms are common in COVID-19 infections (Collantes et al., 2021), and in PASC these can include lingering fatigue, brain fog, or attentional issues that are increasingly being described as neurological-PASC (N-PASC) (Perez Giraldo et al., 2023). N-PASC is increasingly thought to be accompanied by cognitive dysfunction and a decline in attention, working memory, and cognitive throughput (Sekendiz, 2024). Prior work has identified increases in subcortical gliosis in the hippocampus in those with increased depressive symptoms following COVID-19 infection (Braga et al., 2023), while other work has reported that changes to fractional anisotropy and isometric organization are evident in the white matter among individuals with N-PASC (Clouston et al., 2024). Together, these findings have lead researchers to suggest that neuroglial activation might emerge from a cytokine storm that causes glial or macrophagic reactions in the brain (Grant et al., 2024), though findings supporting this interpretation may be biased because many individuals exhibiting symptoms of N-PASC experienced neurological damage during an acute bout of severe COVID-19.

A seminal study found evidence of subcortical gliosis among individuals reporting increased depressive symptoms following COVID-19 infection (Braga et al., 2023), but did not examine variations in neuroinflammation among individuals with one of the most common symptoms of N-PASC – Brain Fog. Changes in translocator protein (TSPO) expression result from glial or macrophagic hyperactivity (Chen et al., 2021) and that cerebral gliosis can therefore be measured using [^18^F]-FEPPA with positron emission tomography (PET). The purpose of this study was to use [^18^F]-FEPPA to examine the severity and distribution of widespread neuroinflammation in the cortex, white matter, or cerebellum, and to determine if cerebral gliosis was more evident in points of entry or vulnerability including in the hippocampus or in the orbitofrontal and medial frontal cortices in N-PASC.

## 2 Methods

### 2.1 Ethics

The Stony Brook University Institutional Review Board approved this study. All participants provided informed written consent at enrollment in the study after study procedures were fully explained. The study followed the Strengthening the Reporting of Observational Studies in Epidemiology (STROBE) reporting guidelines (Von Elm et al., 2007).

### 2.2 Setting

Participants are essential workers who were recruited from September 2022–June 2023 from a clinic-based monitoring program that has been collecting data for the past two decades on workers living in Long Island, NY (Dasaro et al., 2017). This program was proactive in recording the onset of COVID-19 in NY (Morozova et al., 2021), has linked onset and severity to polygenic and clinical risk factors (Waszczuk et al., 2023), and has previously described the development of PASC symptom at midlife in non-hospitalized essential workers (Lhuillier et al., 2022) and linked risk for PASC to vaccination and multiple infections (Babalola, 2025). Due to the occupational inclusion criteria this cohort is predominantly male, and most participants are under the age of 70. Additionally, individuals in the cohort have high lifetime employment levels (>99.5%), high educational attainment (>75% have at least some college education), and approximately half were working at the time of the COVID-19 pandemic.

### 2.3 Inclusion/exclusion

To be included in this study, N-PASC participants were required to have a diagnosis of N-PASC that persisted until the scanning appointment. Controls, on the other hand, were all scanned using the same protocol on the same scanner prior to the COVID-19 pandemic. All study participants lacked a history of psychosis, a history of serious head trauma, stroke, or other neurological disorders, serious medical condition such as renal failure and autoimmune disease, a known history of hepatitis C, any contraindication for PET or MRI scanning (e.g., pacemaker, metallic implants, pregnancy, etc.). To participate in the study, individuals also needed to be fluent in English and have the capacity to provide informed consent. To fit into the PET/MRI scanner, participants had a body mass index at the time of scan ≤40 kg/m^2^. Finally, to complete the PET component we excluded individuals currently using cognitively active medications (e.g., methylphenidate, anticholinergic, or antipsychotic medications) and those using anti-anticoagulant or anti-inflammatory medications. Because [^18^F]-FEPPA binds poorly among individuals who have a relatively rare genotype indicating low affinity for TSPO polymorphism, we excluded individuals whose genotyping indicated this affinity (Mizrahi et al., 2012). *TSPO genotype* was based on the rs6971 polymorphism and was measured using blood samples collected at screening. Following recommended procedures (Kreisl et al., 2013; Owen et al., 2011), all subjects were genetically profiled and classified as high-affinity (HABs, Ala147/Ala147), and mixed-affinity (MABs, Ala147/Thr147). Low-affinity binders (LABs, Thr147/Thr147) were excluded from this study.

### 2.4 N-PASC diagnosis and controls

To be diagnosed with N-PASC, participants first had to prove that they had developed mild to moderate COVID-19 without evidence of neurological complications. Specifically, participants had to report the presence, and describe symptom types and date of onset, for COVID-19 symptoms. Diagnoses were verified using evidence from medical records or reports from valid testing facilities showing a positive COVID-19 polymerase chain reaction (PCR), antibody, or antigen testing. COVID-19 survivors who recovered at home without hospitalization and none had acute neurological complications (Morozova et al., 2021). COVID-19 survivors were diagnosed with *N-PASC* after having reported persistent neurological symptoms of COVID-19, notably new-onset of reported brain fog, lasting at least 6 months after the acute COVID-19 infection had resolved (Thaweethai et al., 2023). To participate in this imaging study, participants had to report symptoms until screening and scanning were completed. We report the months between COVID-19 symptom onset date and the date neuroimaging date as N-PASC duration.

*Pre-COVID-19-Pandemic Controls* were individuals who completed the same scanning protocol immediately prior to COVID-19 pandemic and who were healthy at the time of scanning.

### 2.5 [^18^F]-FEPPA PET/MRI scanning

The outcome of this study was the cerebral distribution volume for Fluorine F-18–labeled *N*-(2-(2-fluoroethoxy)benzyl)-*N*-(4-phenoxypyridin-3-yl)acetamide ([^18^F]-FEPPA) as measured using dynamic PET. [^18^F]-FEPPA is a radioactive ligand that was designed to bind to mitochondrial translocator protein 18-kDa (TSPO), which is most abundantly expressed in M2-activated macrophages and microglia (Zammit et al., 2020), making [^18^F]-FEPPA a particularly valuable radioligand for the study of neuroinflammation in N-PASC.

[^18^F]-FEPPA PET scans were performed using a 3T Siemens Biograph mMR hybrid PET/MRI imaging system (Siemens Healthineers, Knoxville, TN, USA). [^18^F]-FEPPA was synthesized locally following a standard process (Wilson et al., 2008) and then ≤185 MBq of [^18^F]-FEPPA was injected as an intravenous bolus while a participant was in the PET/MRI machine and continuous 3D dynamic PET data were acquired in list-mode for 120 min (Rusjan et al., 2011). To enable kinetic modeling, an automated blood sampling system (Twilite; Swisstrace, Zurich, Switzerland) was used to continuously measure arterial blood radioactivity for the first 6 min of the scan, and manual arterial blood samples were collected at 2.5/7/15/30/60/90/120-min time-points following injection. Tracer and metabolite levels were measured using high-performance liquid chromatography analysis for manual blood draws (Rusjan et al., 2011).

A three-dimensional T1-weighted structural magnetization-prepared rapid gradient echo (MPRAGE) structural MRI was obtained simultaneously with PET data acquisition using the following parameters: TR = 2300 ms, TE = 3.24 ms, TI = 900 ms, flip Angle = 9°, acquisition matrix: 240 × 256, isotropic voxel resolution: 0.87 × 0.87 × 0.87 mm^3^.

### 2.6 Incidental neuroradiological assessment

A trained neuroradiologist conducted examinations of incidental findings by relying on information from anatomical MRI studies including the T1-MPRAGE and T2-FLAIR data.

### 2.7 Distribution volume

The PET images were reconstructed into 21 timeframes according to the binning scheme: 4 × 1min, 3 × 2min, 6 × 5min, 8 × 10min. Raw list-mode PET data was reconstructed offline using Siemens’ e7 Tools software (all quantitative corrections applied including a CT-like MR-based attenuation map (Izquierdo-Garcia et al., 2014; Ladefoged et al., 2017) and filtered back-projection using ramp filter and voxel size of 2.08 mm trans-axial x 2.03 mm axial). Motion correction and PET-to-MRI co-registration were done before time activity curves were generated as previously described (Iscan et al., 2017). To obtain a quantitative measure of [^18^F]-FEPPA uptake, two-tissue-compartmental (2 TC) kinetic modeling was performed to quantify the total distribution volume (V_T_) as the outcome measure, as previously validated (Rusjan et al., 2011). The whole blood to plasma radioactivity ratio data was fit and then used to convert the whole blood automated early arterial sampling measurements to plasma equivalent. These early plasma estimates were then concatenated with the manual arterial plasma draws. To generate the final metabolite-corrected arterial plasma input function for the kinetic analysis, metabolite correction was performed using a Hill function to fit the percentage of unmetabolized tracer, and the resulting input function was fit to a straight line to the peak followed by the sum of three exponentials after the peak. The arterial whole blood curve was used to correct activity in the vascular component, assuming that the blood volume is 5% of the total brain volume (Leenders et al., 1990). To adjust for differences in V_T_ across TSPO genotypes, we multiplied the levels in individuals with the medium affinity binding genotype by 1.37 (Kreisl et al., 2013). Each brain was non-linearly registered to MNI space using the T1 MRI and parcellated into volumes of interest using the Columbia Atlas (cba.nyspi.org). [^18^F]-FEPPA VT was calculated as described above for each volume. We reported data for gross regions including the cerebral cortex, cerebral white matter, hippocampus, amygdala, and whole cerebellum. Researchers have reported that increases in [^18^F]-FEPPA tend to be global across the brain so we did not perform partial volume correction since it is unlikely to have an effect and could increase noise.

*SUV Analyses*: [^18^F]-FEPPA V_T_ is exceedingly difficult and moderately risky to obtain because it requires an arterial line. Thus, we sought to visualize differences using data from the 75-120-min window to calculate standardized uptake values (SUV_75-120_). However, since V_T_ is a dynamic measure that quantifies the total lifetime distribution volume, we calculated a similar value using dynamic SUV values collected across the timeframes described above, relying on the 10-120-min time-window to calculate DSUV = $\int_{0}^{\infty }\;\ln\left({SUV}_{t}\right)$. Regional DSUV was moderately associated with regional V_T_ values in the cerebellum (rho = 0.60, p = 0.041), orbital prefrontal cortex (rho = 0.46, p = 0.039), medial prefrontal cortex (rho = 0.45, p = 0.047), hippocampus (rho = 0.46, p = 0.041) gray matter (rho = 0.47, p = 0.036) but not in the white matter (rho = −0.11, p = 0.636). V_T_ was not associated with SUV_75-120_ in any region (p-values ranging from 0.450 to 0.935).

*Regions of Interest:* We opted to rely on gross anatomical regions including the gray matter, white matter, cerebellum, and hippocampus. We also incorporated the medial prefrontal cortex and orbitofrontal cortex because these cortices lie above the olfactory centers and behind the ocular nerves and might be neuroinvasive points of entry. Finally, we included the anterior cingulate and ventral and dorsal striatum to be consistent with (Braga et al., 2023).

### 2.8 Non-COVID symptomatologic measures

*Premorbid Cognitive Ability* is meant to indicate cognitive performance in the context of potential neurocognitive disease and was measured using the wide range achievement test and standardized scores were reported (mean = 100, SD = 15). *Global Cognition* was measured in all studies using the Montreal Cognitive Assessment (Nasreddine et al., 2005). To examine different domains of executive functioning that we felt were likely to reflect changes due to cerebral infections, we measured: 1) verbal fluency, as measured using the controlled word association test (Clouston et al., 2024); 2) working memory, measured using the symbol-digits modalities test (Benedict et al., 2017), and 3) executive function was estimated using the difference between the Trails B and Trails A tests (Feeney et al., 2016), a validated measure of executive function (Llinàs-Reglà et al., 2015).

*Pre/Post-COVID-19 Depressive symptoms* were collected using the nine-item patient health questionnaire (PHQ-9) and, to facilitate comparison, we reported both their post-COVID depressive symptoms, matched with pre-COVID depressive symptoms using data for all N-PASC subjects to data retrieved from pre-pandemic monitoring data. Finally, we calculated the pre/post-COVID differences in depressive symptoms using the subtraction of post-COVID PHQ-9 – pre-COVID PHQ-9 scores.

### 2.9 Statistical analyses

Descriptive statistics and violin plots were reported for the whole sample and stratified by the presence of N-PASC. Because V_T_ was not readily visualized, we relied on mean SUV_75-120_ to generate cerebral maps for visual comparison. Bivariate analyses relied on Welch's *t*-test because of the potential for unequal variance and unequal sample sizes between groups; we reported standardized mean differences.

Due to the high potential for non-Gaussian data for V_T_ outcomes, we also reported information from analysis of variance (ANOVA). When examining correlations between V_T_ and cognitive or behavioral symptoms, we relied on Spearman's rho (ρ). Bivariable and multivariable statistics also examined group differences in global and regional [^18^F]-FEPPA V_T_ relying on generalized linear models (GLM) to fit a model using a Wald distribution (Inverse-Gaussian) model. Wald models were used to model V_T_ over other models including the linear regression model because V_T_ reflects a count-like data generation process causing positive datapoints with severe skew (Deri et al., 2021). Log-Wald models were used to report distribution ratios (DR) and accompanying 95% confidence intervals.To ensure that our modeling distribution was valid, we conducted fit analyses using GLM to compare between models using four potential distributional assumptions, and the Bayesian Information Criterion (BIC) was reported to facilitate model comparison. Multivariable analyses included adjustment for age, gender, and genotype status (HAB *versus* MAB) to adjust for the effect of TSPO *rs6971* polymorphism on radioligand binding affinity. Multivariable-adjusted secondary analyses examined the main effects of the associations between [^18^F]-FEPPA binding in each ROI and performances across cognitive domains.

To compare between measures and to help understand the interpretation of these results, we reported the area under the receiver-operating curve (AUC). Since a cutoff is usually required to determine positive/negative status, we used Youden's index to create a valid cutoff for cerebral ROIs and report sensitivity, specificity, negative predictive value, false positive rate, accuracy, and the F1-score at the optimal cutoffs. Throughout the analyses, we use two-tailed p-values to determine statistical significance (α = 0.05), and also reported when statistical tests passed the false discovery rate (FDR = 0.05) (Benjamini et al., 1995). Statistical analyses were completed using Stata 17/MP [StataCorp].

## 3 Results

In total, ten essential workers with N-PASC went through the [^18^F]-FEPPA imaging study. Sample characteristics of the whole sample and stratified by COVID-19 diagnostic status (Table 1). Incidental review identified one participant with one small chronic infarction in the left cerebellum that is believed, based on a careful chart-review to predate COVID-19 infection. Sensitivity analyses showed that excluding this individual from further analysis showed no substantively important differences in results, so this individual was retained in all analyses. In this table, we found that the pre-pandemic group had lower BMI and appeared younger than the N-PASC group. The N-PASC group had much higher post-COVID-19 depressive symptoms. However, there were no differences in terms of TSPO genotype, premorbid cognitive ability, or sex. We recorded acute symptoms to help describe the sample, but since no symptoms were associated with any measure of neuroinflammation we did not report these results. However, arterial line placement was unsuccessful for two participants, resulting in V_T_ being available for eight participants. While we were able to estimate V_T_ for eight participants, analyses showed no statistically significant differences in DSUV or SUV_75-120_ among those with N-PASC with versus without V_T_ (p-values ranged from 0.162 to 0.714).

**Table 1:** Sample characteristics for people who completed the [^18^F]-FEPPA neuroimaging study.

Characteristic	Neurological Post-Acute Sequelae of COVID-19 (n = 10)	Pre-COVID-19 Pandemic Controls (n = 14)	P
Age, years	61.1 (5.0)	56.4 (5.6)	0.04
Body Mass, kg/m^2^	33.1 (3.0)	30.3 (3.7)	0.06
Pre-Pandemic Depressive Symptoms	5.5 (5.0)	3.0 (3.1)	0.14
Global Cognition	23.0 (3.7)	25.2 (3.4)	0.14
Verbal Fluency	34.1 (9.5)	36.2 (7.7)	0.36
Working Memory	49.5 (9.2)	40.6 (12.1)	0.07
Executive Function	57.6 (44.7)	45.9 (18.3)	0.38
Premorbid Cognitive Ability	97.6 (12.5)	93.1 (7.8)	0.34
Female	2 (20%)	2 (14.3%)	0.71
TSPO Genotype HAB *versus* MAB	4 (40%)	6 (42.9%)	0.89

Moderate *versus* Mild Acute COVID-19	7 (70%)	–	
Vaccinated at time of Neuroimaging	9 (90%)	–	
Months Since COVID-19 Infection	22.2 (14.4)	–	
Post-COVID Depressive Symptoms	8.3 (4.6)	–	
Difference between Pre- vs. Post-COVID Depressive Symptoms	4.6 (2.6)	–	

Most Common Acute COVID-19 Symptoms			
Fever	9 (90%)	–	
Joint or Muscle Pain	8 (80%)	–	
Cough	8 (80%)	–	
Headache	7 (70%)	–	
Shortness of Breath	7 (70%)	–	

**Note**: ∗Comparing N-PASC group results to their own pre-COVID-19 depressive symptom scores. Summary statistics are presented as Mean (SD) or N (%). TSPO genotype was grouped into high-affinity binder and medium-affinity binder groups. Low-affinity binding individuals were excluded from this study. P-values were estimated using non-parametric trend tests.

**Fig. 1:**
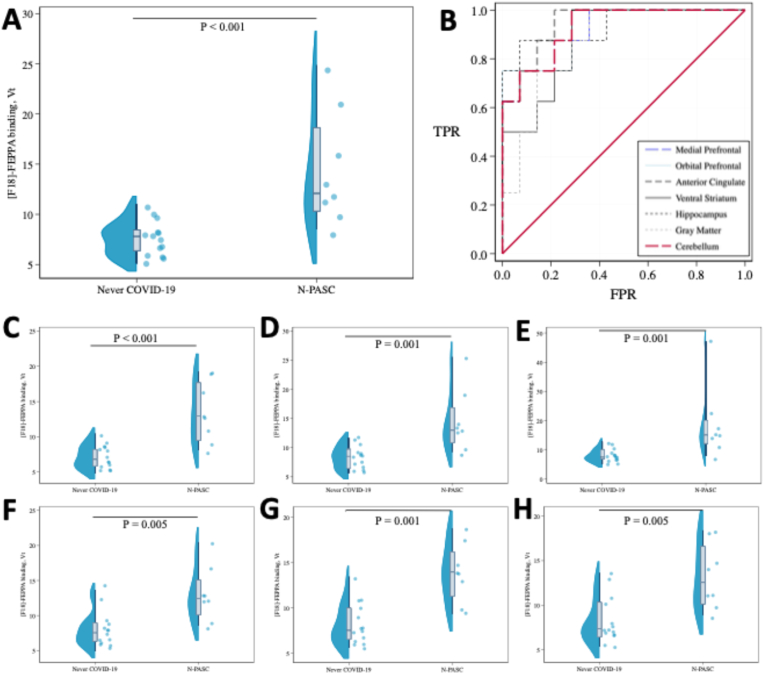
Violin Plot showing cortical [^18^F]-FEPPA distribution volume (Vt) in 22 essential workers, comparing N-PASC versus Pre-Pandemic controls and stratified by cerebral region: **A:** Anterior Cingulate; **B:** Receiver Operating Curves for N-PASC when examining [^18^F]-FEPPA binding in the strongest four cerebral regions; **C:** Medial Prefrontal Cortex; **D:** Orbital Prefrontal Cortex; **E:** Hippocampus; **F:** Ventral Striatum; **G:** Cerebellum. **H:** Gray Matter, **H:** All p-values shown are statistically significant after adjusting for the false discovery rate (FDR = 0.05).

We present differences in V_T_ between groups in Fig. 1. Note that results appear abnormal in all violin plots, and that summary statistics show high kurtosis for V_T_ values including, for example, hippocampal gliosis (k = 2.0). Each of the figures (Fig. 1A–E) show large, statistically significant, differences between N-PASC and never-COVID-19 controls. The receiver operating curve (Fig. 1F) all show strong associations between V_T_ and N-PASC.

As shown in Table 2, these studies revealed elevations in hippocampal V_T_ (mean = 18.58) as compared to 7.97 in pre-pandemic controls, a difference of 10.62 mL/cm^3^. As estimated by log-Wald models, this represents a 1.70-fold (95% C.I. = [1.30–2.21]) increase (p = 0.001) in the [^18^F]-FEPPA-V_T_. We saw similar increases in the medial prefrontal cortex (13.3 versus 7.1, DR = 1.88 [1.46–2.44] P < 0.001), orbital prefrontal cortex (14.39 versus 8.27, DR = 1.74 [1.33–2.28] P = 0.001), gray matter (12.90 versus 7.91, DR = 1.693 [1.21–2.20] P = 0.005), cerebellum (13.75 versus 8.10, DR = 1.68 [1.13–2.48] P = 0.001), and white matter (13.48 versus 8.03, DR = 1.68 [1.10–2.79] P = 0.019).

**Table 2:** Unadjusted and multivariable-adjusted associations between the presence of N-PASC and [F^18^]-FEPPA binding in cerebral subregions, stratified by distributional assumptions made across statistical models.

Difference	N-PASC	Never-COVID-19	ANOVA		Welch's *t*-test		Log-Gamma Regression	
	Mean (SD)	Mean (SD)	F	P	Cohen's D	P	DR [95% C.I.]	P
**Hippocampus**	**18.75 (12.28)**	**8.07 (2.2)**	**10.39**	**0.004**	**1.19**	**0.043**	**1.69 [1.30**–**2.20]**	**0.001**
**Medial Prefrontal Cortex**	**13.45 (4.38)**	**7.17 (1.69)**	**23.46**	**<0.001**	**1.49**	**0.004**	**1.88 [1.45**–**2.42]**	**<0.001**
**Orbital Prefrontal Cortex**	**14.52 (5.43)**	**8.37 (1.96)**	**15.04**	**0.001**	**1.33**	**0.014**	**1.74 [1.33**–**2.26]**	**0.001**
**Anterior Cingulate**	**14.43 (5.85)**	**7.57 (1.74)**	**17.19**	**<0.001**	**1.38**	**0.012**	**1.91 [1.44**–**2.51]**	**<0.001**
**Ventral Striatum**	**13.23 (3.76)**	**8.25 (2.53)**	**13.87**	**0.001**	**1.30**	**0.006**	**1.60 [1.20**–**2.14]**	**0.005**
**Dorsal Striatum**	**34.3 (69.12)**	**20.4 (50.78)**	**0.29**	**0.594**	**0.24**	**0.628**	**1.68 [0.17**–**16.85]**	**0.664**
**Gray Matter**	**13.02 (4.00)**	**8 (2.52)**	**13.19**	**0.002**	**1.28**	**0.008**	**1.63 [1.21**–**2.19]**	**0.005**
**White Matter**	**13.6 (6.95)**	**8.12 (2.9)**	**6.81**	**0.017**	1.02	0.063	**1.67 [1.14**–**2.46]**	**0.018**
**Cerebellum**	**13.89 (3.28)**	**8.2 (2.32)**	**20.90**	**0.000**	**1.45**	**0.001**	**1.69 [1.29**–**2.20]**	**0.001**

**Note**: Statistically significant results are shown in bold typeface for ease. P-values determined by student's t-tests. ANOVA: analysis of variance; Welch's *t*-test adjusts for an assumption of unequal variance. DR: Distribution volume ratio estimated using Wald's log-inverse-Gaussian regression. 95% C.I.: 95% confidence interval.

**Fig. 2:**
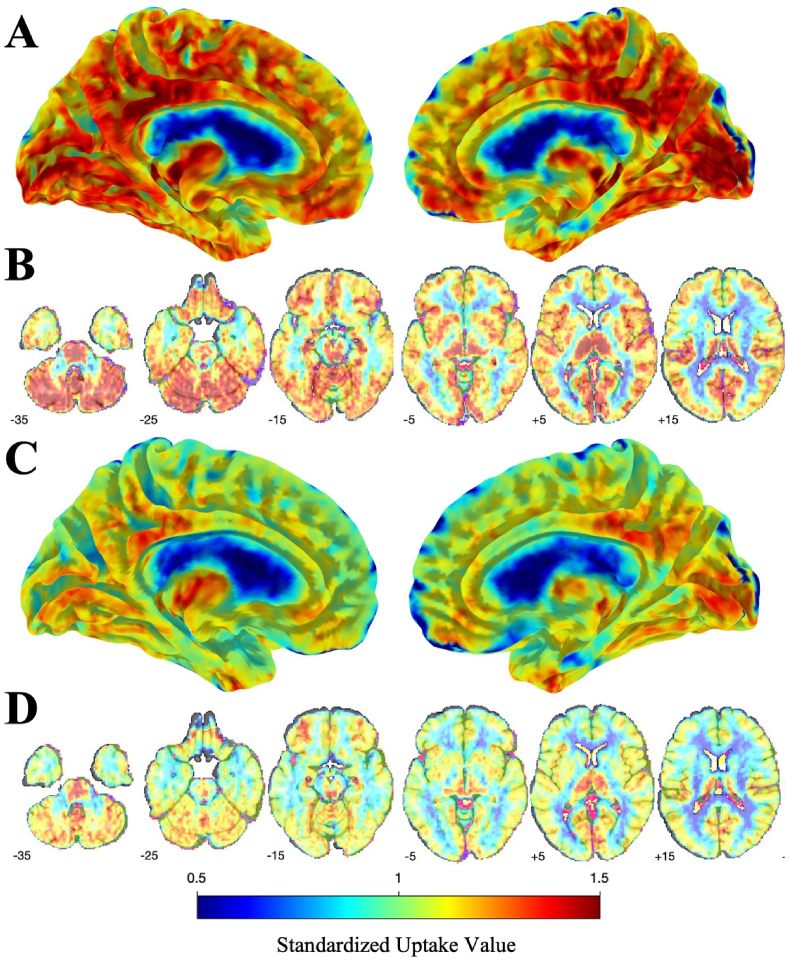
Fused [^18^F]-FEPPA and Structural Image showing differences by standardized uptake values (SUV) in participants with persistent Brain Fog following COVID-19 infection as compared to pre-pandemic controls. Panel A shows TSPO SUV in N-PASC + participants, mapped to the cerebral surface. Panel B shows [^18^F]-FEPPA SUV values mapped to the cerebral parenchyma. Panel C shows [^18^F]-FEPPA SUV values for pre-pandemic controls mapped onto the cerebral surface. Panel D shows similar values for [^18^F]-FEPPA SUV values among pre-pandemic controls throughout the cerebral parenchyma.

To examine the cerebral distribution of differences in V_T_ we next provided an average image using SUV_75-120_ to visualize the regional TSPO distribution across the cerebrum (Fig. 2, Panels A–D) as well as in the cerebellum though results from the white matter appeared mild (Fig. 2, Panels B and D). This figure revealed that [^18^F]-FEPPA uptake in the cortex was broadly associated with N-PASC. Differences in the gray matter SUV_75-120_ were only evident when relying on log-Wald regression models controlling for TSPO genotype (p = 0.035), but not when using ANOVA (p = 0.086) or Welch's t-tests (p = 0.104). Using gray matter DSUV revealed statistically significant differences when using log-Wald models (p < 0.001), ANOVA (p = 0.017), though not when using Welch's t-tests (p = 0.052).

Examining differences among in-sample AUC statistics (Table 3) revealed that [^18^F]-FEPPA V_T_ was highly capable of differentiating N-PASC from pre-pandemic controls. Additionally, we found that measures of DSUV were also reliably capable of differentiating N-PASC from pre-pandemic controls though with lower overall reliability across regions. However, we found that while AUCs for SUV_75-120_ values trended in the same direction, they were generally too weak to reliably identify differences in this study. Overall, results shown in the AUC analyses are replicated and supported by additional measures of accuracy contained in eTable 1. Notably, the sensitivity/specificity of hippocampal [^18^F]-FEPPA distribution volume (0.88/0.93 respectively) was exceptionally high. The F1-score ranged from a low of 0.75 when using the white matter to a high of 0.88 in the hippocampus.

**Table 3:** Area under the receiver-operating curve (AUC) showing the ability for cerebral regions of interest to accurately identify individuals with Neurological Post-Acute Sequelae of Coronavirus Disease (2019

Region of Interest	V_T_	V_T_ (excluding max value)	VSUV	SUV_75-120_
	AUC [95% C.I.]	AUC [95% C.I.]	AUC [95% C.I.]	AUC [95% C.I.]
Hippocampus	**0.95 [0.85**–**1.00]**	**0.93 [0.8**–**1.00]**	**0.81 [0.59**–**1.00]**	0.61 [0.36–0.87]
Medial Prefrontal	**0.93 [0.82**–**1.00]**	**0.91 [0.78**–**1.00]**	**0.81 [0.6**–**1.00]**	0.68 [0.42–0.93]
Orbital Prefrontal	**0.93 [0.82**–**1.00]**	**0.92 [0.79**–**1.00]**	**0.80 [0.59**–**1.00]**	0.63 [0.36–0.89]
Anterior Cingulate	**0.95 [0.86**–**1.00]**	**0.94 [0.84**–**1.00]**	**0.67 [0.43**–**0.92]**	0.68 [0.42–0.93]
Ventral Striatum	**0.81 [0.63**–**0.99]**	**0.87 [0.71**–**1.00]**	**0.74 [0.51**–**0.97]**	0.63 [0.38–0.87]
Dorsal Striatum	**0.88 [0.75**–**1.00]**	**0.79 [0.59**–**0.99]**	**0.62 [0.38**–**0.87]**	0.58 [0.34–0.83]
Gray Matter	**0.86 [0.7**–**1.00]**	**0.85 [0.68**–**1.00]**	**0.78 [0.55**–**1.00]**	0.72 [0.47–0.97]
White Matter	**0.84 [0.66**–**1.00]**	**0.82 [0.62**–**1.00]**	0.65 [0.37–0.92]	0.67 [0.40–0.93]
Cerebellum	**0.92 [0.81**–**1.00]**	**0.92 [0.8**–**1.00]**	**0.82 [0.61**–**1.00]**	0.69 [0.42–0.96]

**Note**: AUC: area under the receiver operating curve; V_T_: distribution volume; SUV: standardized uptake value; DSUV: dynamic SUV. Results where the confidence interval around the AUC did not include 0.50.

**Fig. 3:**
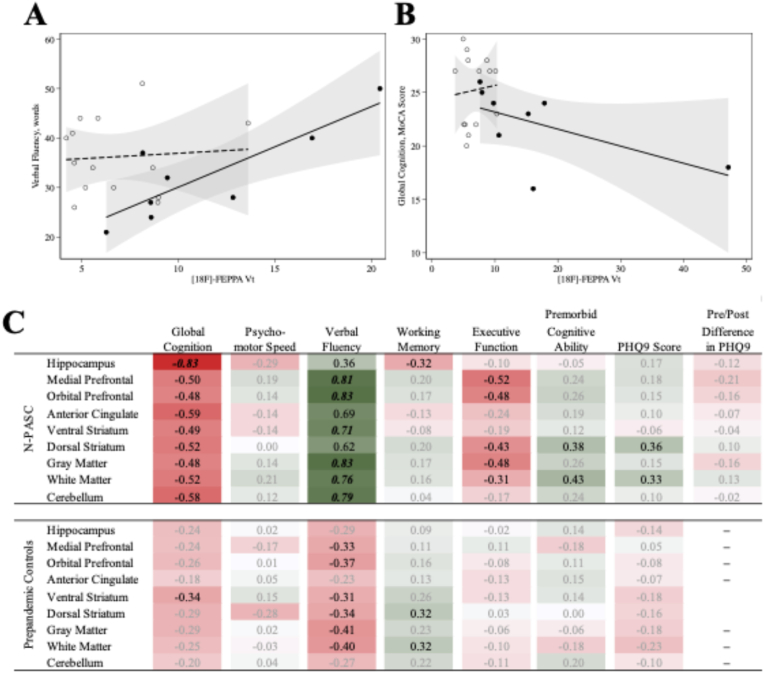
Associations between regional [^18^F]-FEPPA V_T_ and cognitive function and depressive symptoms in participants with and without Neurological Post-Acute Sequelae of COVID-19: A) scatter and overlaid linear plots examining association between Gray Matter [^18^F]-FEPPA V_T_ and Verbal Fluency, stratified into N-PASC + participants (black dots, solid line) and pre-pandemic controls (empty dots, dashed line); B) scatter and overlaid linear plots examining association between Hippocampal [^18^F]-FEPPA V_T_ and Global Cognition, stratified into N-PASC + participants (black dots, solid line) and pre-pandemic controls (empty dots, dashed line); C) full correlation results reporting Spearman's rho describing associations between regional [^18^F]-FEPPA Binding (Vt) with all measures of cognitive function and depressive symptoms among participants with Neurological Post-Acute Sequelae of COVID-19. Results for all reasons shown, results with moderate correlations (||rho||>0.30) are shown in black typeface. Statistically significant (p < 0.05) correlations are shown using ***bold italics***.

We examined the association between [^18^F]-FEPPA binding and cognitive symptoms among individuals with N-PASC (Fig. 3). We found consistent trends when examining associations between V_T_ and improved cognitive performance, though only the strongest results linking V_T_ with higher verbal fluency were statistically significant. Intriguingly, all statistically significant results were isolated to the N-PASC group, but associations with verbal fluency appeared to go in the opposite direction across both groups while results for global cognition the appeared to be in the same direction across both groups though muted in the prepandemic control group. Sensitivity analyses examining associations between PASC duration and V_T_ found a statistically significant decrease in White Matter V_T_ by duration (DR = 0.85 [0.77–0.94] P = 0.0001) that was not replicated when examining DSUV (p = 0.522) or SUV_75-120_ (p = 0.442).

To understand the impact of adjusting for covariates we examined the potential for analytic bias due to differences in the distributional sensitivity of different models relying on various modeling assumptions revealed that the log-Wald model had the lowest BIC value and was therefore deemed to be a superior fit to alternatives (eTable 2). Unadjusted and multivariable-adjusted results from log-Wald models showed that adjustment for age and body mass increased the effect size attributed to each association.

## 4 Discussion

In the first case-control study to assess cerebral gliosis in people reporting persistent brain fog due to N-PASC, we found evidence of the presence of widespread cortical and subcortical gliosis in a cohort of essential workers who reported acute mild to severe COVID-19 and lingering symptoms consistent with N-PASC. Our study revealed that symptoms indicating N-PASC were associated with substantial increases in glial activation across the cerebrum with focal points in the hippocampus, gray matter, and cerebellum even among essential workers who survived mild to moderate COVID-19 infections that did not require hospitalization. Results from this study add to a growing literature describing neuroinflammation as a central aspect of Neurological PASC (N-PASC). Together with other studies that also support the view that neuroimaging and TSPO imaging in particular can effectively discriminate N-PASC, this study suggests that N-PASC may be verified using neuroimaging-focused biomarkers including [^18^F]-FEPPA distribution volume.

Diagnostic verification for the presence of any syndrome, and N-PASC in particular, is critical to verifying diagnosis and to showing treatment effectiveness. Therefore, the accuracy of biomarkers reported in this study and in other similar studies is increasingly necessary. For example, in the present study the AUC for hippocampal [^18^F]-FEPPA distribution volume adjusted for TSPO genotype was 0.95 [0.85–1.00]. Model fit estimates for hippocampal gliosis overall and when relying on cutoffs (shown in eTable 1) revealed that when compared to healthy controls, hippocampal [^18^F]-FEPPA distribution volume was able to identify individuals with N-PASC with sensitivity/specificity/F1 at the estimated cutoff of 0.88/0.93/0.88. If replicated, this degree of accuracy could support the use of [^18^F]-FEPPA to positively support a diagnosis of N-PASC.

This work expands upon prior work finding subcortical gliosis in those with increased depressive symptoms following COVID-19 infection (Braga et al., 2023). Our results are comparable with that study in evaluating hippocampal gliosis using similar methods including reliance on pre-COVID-19 controls (Braga et al., 2023). However, wherein the authors reported that hippocampal [^18^F]-FEPPA binding was increased by 1.51 mL/cm^3^ (when compared to 7.72, an increase of 19.6%) in those with delirium and headaches after COVID-19 whereas the present study reported an increase of 5.2 mL/cm^3^ (compared to 6.88 mL/cm^3^ among pre-pandemic controls, this is an increase of 75.6%) in the cerebellum, and 4.7 mL/cm^3^ increase in the gray matter (compared to 6.73 mL/cm^3^ among pre-pandemic controls, this is an increase of 69.1%) among those with brain fog after COVID-19. Our results are also consistent with results reported in a case series (n = 2) using [^18^F]-DPA-714 to measure microglial activation to find a two-fold increase in binding potential (Visser et al., 2022). While this study does support prior work, this study recruited people with brain fog rather than those with depression, potentially suggesting that the inflammatory pathway is affected by the presence of brain fog. Other differences may include differences in the strain of the disease, the severity of COVID-19 infection, or the types and availability of treatment.

The present work supports a growing number of structural and connective neuroimaging studies reporting findings consistent with neuroinflammation in people with N-PASC. For example, work using magnetic resonance imaging has reported lower post-recovery axonal density (Huang et al., 2022) and changes in the cerebral connectome consistent with neuroinflammation among hospitalized patients with severe COVID-19 (Rau et al., 2022). Additionally, previous studies have reported similar findings to pervade individuals from the general population who reported mild-to-moderate COVID-19 (Petersen et al., 2023) and essential workers with N-PASC (Clouston et al., 2024). Our results support these findings but further suggest that the role of neuroinflammation may not always be maladaptive in N-PASC.

We reported that gliosis was most consistently associated with COVID across the medial and orbital portions of the frontal lobe. The frontal lobe is where COVID-19 is most likely to invade the cerebrum through the olfactory bulb (Meinhardt et al., 2021). Interestingly, while the medial and orbital prefrontal cortex lies above the eyes and the olfactory bulb, making them potential points of neuroinvasion, this is not true of the hippocampus where we found the strongest regional results. Neuroinflammation in this study was widespread throughout the cortical and subcortical structures and in the cerebellum. Coupled with evidence of improved cognition in people with N-PASC these results might indicate the presence of a robust neuroimmune response in these regions is associated with improved outcomes.

There is concern that inflammation could be damaging the cerebrum in N-PASC. We found that while [^18^F]-FEPPA was increased in N-PASC, individuals whose [^18^F]-FEPPA V_T_ was higher seemed to have better verbal fluency than those with more normal [^18^F]-FEPPA binding levels. This result might imply that the neuroinflammation seen in N-PASC is supporting an adaptive immunological response, which is an unanticipated result if the brain is experiencing a singular systemic shock but may be expected in the case that there is a viral reservoir as has been hypothesized to occur in N-PASC (Proal et al., 2023). Yet, the same data suggested that global cognitive performance as measured using the Montreal Cognitive Assessment was reduced in those individuals with higher hippocampal [^18^F]-FEPPA distribution volume. These results support the view that reductions in cerebral gliosis might emerge when the cerebrum is damaged, a process that might specifically reduce global cognitive performance.

SARS-CoV-2 has continued to evolve rapidly since it began infecting individuals and has continued to mutate in order to improve transmissibility (Markov et al., 2023). Most of these participants are essential workers who developed PASC on their first infection prior to the widespread availability of vaccines. However, different variants have different symptoms and vary in terms of severity and the risk of PASC. For example, as one large study showed the risk of PASC was much lower following infection with omicron versus delta and pre-delta variants (Xie et al., 2024), while some results imply that omicron subvariants also differ in terms of severity (Klein et al., 2023). Overall, this literature implies that the risk of PASC is lower now that more people have been vaccinated amd are exposed to omicron-based variants, these mutations have also allowed the virus to both re-infect individuals who have been previously infected and break through protections offered by vaccination. Thus, while the risk of N-PASC may be lower, now, than in the first years of the pandemic the risk of being re-infected increases yearly and that also increases the risk of follow-up N-PASC.

Inconsistencies between results and expectations may result from multiple reasons. The first is the phase-specific nature of the neuroimmune system, which relies on several types of astrocytes and cerebral macrophages that all have pro- and anti-inflammatory phases (Spiteri et al., 2022). While macrophages do not express translocator protein (TSPO) to the same extent as glial cells, TSPO ligands seem to attach to different cell types and during different cell phases. To our knowledge, [^18^F]-FEPPA tends to bind to M2 glial cells (Zammit et al., 2020), while other ligands may indiscriminately target TSPO-expressing cells (Chen et al., 2021). Our results could suggest that the glial activation shown may be partially due to the need for the cerebrum to either clear out lingering viral particles or repair damage initially caused by the COVID-19 infection. Concurrently, TSPO could also be expressed by other cell types in the neuroimmune environment, such as astrocytes and vascular endothelial cells (Guilarte et al., 2022). This possibility is consistent with the role of these type of cells in repairing damage caused by COVID-19 to the endothelial and neurovascular structures, as noted in the other studies (VanElzakker et al., 2024). The second reason for our findings could be fact that the TSPO protein, even if expressed by the microglia, seems to have different functions. In fact, meanwhile TSPO is clearly associated with pro-inflammation activated microglia cells in mice, recent studies suggest that while TSPO is an accurate indicator of microglial cell density that it may not be a clear indicator of pro-inflammatory activation (Nutma et al., 2023). This strengthens the possible view that the microglial cells, whose presence we found to be increased, might protect verbal fluency in N-PASC. Future work should determine whether monocytic response mechanisms and endothelial cell functioning, which uphold macrophagic reactivity, are also active and adaptive in N-PASC.

In this study, we did not report a standardized uptake value ratio (SUVR) because gliosis occurs throughout the brain and there is no natural reference region, making comparison with recent studies difficult (VanElzakker et al., 2024). For example, we found here that differences in V_T_, DSUV, and to a lesser extent in SUV_75-120_ in the white matter and, more importantly, in the cerebellum that were also elevated in COVID-19 and would render most SUVR analyses ineffectual. However, in hopes of providing a visualization we calculated both the SUV_75-120_ and dynamic SUV (DSUV) calculated using a process akin to the one used to calculate the gold-standard distribution volume (V_T_) values. In these data, DSUV were moderately correlated with V_T_ values (ρ = 0.42 in the gray matter and 0.60 in the cerebellum) and these studies provided mildly weaker results to those found using V_T_. For example, while SUV_75-120_ values were unable to identify group differences between N-PASC and pre-pandemic controls, DSUV values were able to more reliably detect a difference. DSUV values benefit from being quantifiable without an arterial line and might therefore help to make future work using TSPO ligands easier since these ligands lack a natural reference region.

### 4.1 Strengths and limitations

We present results from a relatively small study of glial activation at midlife in essential workers who report lingering brain fog after COVID-19 infection. First, while the focus of this study on a sample of essential workers is an important strength of the study, because many individuals were infected by COVID-19 during work before vaccines became available. However, this group is predominantly made up of males at midlife with mild to moderate COVID-19 and is therefore not representative of the general population. While not a central part of this study, we did not find that the women in this study had differences in [^18^F]-FEPPA binding in general or among those with N-PASC supporting the view that these results may be generalizable to others affected by mild to moderate COVID-19. Second, the use of an arterial line is a major limitation because it screens participants who cannot tolerate it and cannot be repeatedly placed thereby reducing the chances that a recruited participant will complete the study, so this study benefited from secondary validation using DSUV. Third, this study is limited because while all N-PASC participants reported symptoms, the clinic from which these participants were recruited was not a PASC clinic so the results from this study may be different from participants who do attend PASC clinics. Fourth, since this study was done in an outpatient setting and included healthy essential workers without acute disease, we did not collect of cerebrospinal fluid to help in assessing COVID-19 biomarkers in hopes of increasing study feasibility. Since [^18^F]-FEPPA has been previously validated in a variety of neurological settings, this only limits our ability to additionally assess the COVID-19 specific inflammatory proteins that might be implicated in N-PASC.

## 5 Conclusions

The increased presence of glial activation in N-PASC when compared to pre-pandemic controls imply that a neuroimmune response is a substantial component of the N-PASC phenotype. Moreover, N-PASC could be a chronic form of autoimmune encephalitis (Graus et al., 2016), though some evidence that verbal fluency was higher in those with more glial activation as well as the lack of recent cerebral hyperintensities generally reported in autoimmune encephalitis. If adaptive, attempts to reduce glial activation could worsen N-PASC prognosis. Concurrently, results from this study might also support some recent work suggesting that there may be a COVID-19 reservoir among individuals with N-PASC (Hanson et al., 2022). Antiviral medications could improve N-PASC prognosis, though little is known about the extent to which such medications cross the blood-brain barrier or might affect glial activation. Thus, while this study does not clarify intervention targets this study does support the view that symptoms of brain fog in N-PASC represent significant increases in glial activation.

## CRediT authorship contribution statement

**Sean A.P. Clouston:** Writing – review & editing, Writing – original draft, Visualization, Validation, Supervision, Software, Resources, Project administration, Methodology, Investigation, Funding acquisition, Formal analysis, Conceptualization. **Paul Vaska:** Writing – review & editing, Visualization, Supervision, Software, Resources, Project administration, Methodology, Formal analysis, Data curation, Conceptualization. **Tesleem Babalola:** Writing – review & editing, Writing – original draft, Validation, Investigation, Data curation, Conceptualization. **John Gardus:** Writing – review & editing, Software, Resources, Methodology, Formal analysis, Data curation. **Chuan Huang:** Writing – review & editing, Supervision, Methodology, Investigation, Conceptualization. **Nicola Soriolo:** Writing – review & editing, Writing – original draft, Project administration, Conceptualization. **Ashley Fontana:** Writing – review & editing, Supervision, Resources, Project administration, Data curation. **Christine DeLorenzo:** Software, Resources, Project administration, Methodology, Formal analysis, Data curation. **Ramin Parsey:** Writing – review & editing, Supervision, Resources, Data curation, Conceptualization.

## Declaration of competing interest

The authors declare the following financial interests/personal relationships which may be considered as potential competing interests: Sean Clouston reports financial support was provided by 10.13039/100000049National Institute on Aging. Benjamin Luft reports financial support was provided by 10.13039/100000125National Institute for Occupational Safety and Health. If there are other authors, they declare that they have no known competing financial interests or personal relationships that could have appeared to influence the work reported in this paper.

## Data Availability

The data that support the findings of this study are available on request from the corresponding author. The data are not publicly available due to privacy or ethical restrictions.
