# Supplementary material for: Glial activation among individuals with neurological post-acute sequelae of coronavirus disease 2019: A positron emission tomography study of brain fog using [18F]-FEPPA
Source: Brain Behav Immun Health. 2025 Jan 16;44:100945. doi: 10.1016/j.bbih.2025.100945 (PMC11786203; doi:10.1016/j.bbih.2025.100945)
Supplement: Multimedia component 1 [file mmc1.docx]

**eTable 1.** Precision and Accuracy Measures to determine the presence of Neurological post-acute sequelae of Coronavirus disease 2019

| **Region** | **Hippocampus** | **Medial Prefrontal Cortex** | **Orbital Prefrontal Cortex** | **Anterior Cingulate** | **Ventral Striatum** | **Dorsal Striatum** | **Gray Matter** | **White Matter** | **Cerebellum** |
| --- | --- | --- | --- | --- | --- | --- | --- | --- | --- |
| Youden's Cutoff | 10.575 | 9.664 | 11.871 | 8.489 | 8.532 | 8.197 | 8.360 | 9.670 | 9.346 |
| Sensitivity | 0.88 | 0.75 | 0.75 | 1.00 | 1.00 | 0.88 | 1.00 | 0.75 | 1.00 |
| Specificity | 0.93 | 0.93 | 1.00 | 0.79 | 0.71 | 0.71 | 0.64 | 0.86 | 0.71 |
| Precision | 0.88 | 0.86 | 1.00 | 0.73 | 0.67 | 0.64 | 0.62 | 0.75 | 0.67 |
| Negative Predictive Value | 0.93 | 0.87 | 0.87 | 1.00 | 1.00 | 0.91 | 1.00 | 0.86 | 1.00 |
| False Positive Rate | 0.07 | 0.07 | 0.00 | 0.21 | 0.29 | 0.29 | 0.36 | 0.14 | 0.29 |
| False Negative Rate | 0.13 | 0.25 | 0.25 | 0.00 | 0.00 | 0.13 | 0.00 | 0.25 | 0.00 |
| Accuracy | 0.91 | 0.86 | 0.90 | 0.86 | 0.82 | 0.77 | 0.77 | 0.82 | 0.82 |
| F1 Score | 0.88 | 0.80 | 0.86 | 0.84 | 0.80 | 0.74 | 0.76 | 0.75 | 0.80 |
| Matthew's Correlation Coefficient | 0.80 | 0.70 | 0.81 | 0.76 | 0.69 | 0.57 | 0.63 | 0.61 | 0.69 |

**eTable 2.** Fit statistics comparing generalized linear models under different distributional assumptions for the unadjusted models comparing the presence of N-PASC and [F^18^]-FEPPA binding in the cerebral gray matter

| **Distribution** | **Bayesian Information Criterion** |
| --- | --- |
| Gaussian | 134.50 |
| **Log-Wald (Inverse-Gaussian)** | **-61.62** |
| Log-Gaussian | -55.29 |
| Log-Poisson | -43.71 |
| Log-Negative Binomial | -60.18 |
| Log-Wald | -60.01 |

**Note**: The best-fitting distributional model for generalized linear modeling has the lowest BIC and is shown using bold typeface.
